# Sodium Benzoate Delays the Development of *Drosophila melanogaster* Larvae and Alters Commensal Microbiota in Adult Flies

**DOI:** 10.3389/fmicb.2022.911928

**Published:** 2022-06-22

**Authors:** Yuling Dong, Zhongfeng Ding, Linxia Song, Desheng Zhang, Changjian Xie, Shujing Zhang, Ling Feng, Hongliang Liu, Qiuxiang Pang

**Affiliations:** ^1^Institute for Anti-aging and Regenerative Medicine Research, School of Life Sciences and Medicine, Shandong University of Technology, Zibo, China; ^2^School of Chemistry and Chemical Engineering, Shandong University of Technology, Zibo, China

**Keywords:** sodium benzoate, *Drosophila melanogaster*, development, commensal microbiota, endocrine hormone

## Abstract

Sodium benzoate (SB), the sodium salt of benzoic acid, is widely used as a preservative in foods and drinks. The toxicity of SB to the human body attracted people’s attention due to the excessive use of preservatives and the increased consumption of processed and fast foods in modern society. The SB can inhibit the growth of bacteria, fungi, and yeast. However, less is known of the effect of SB on host commensal microbial community compositions and their functions. In this study, we investigated the effect of SB on the growth and development of *Drosophila melanogaster* larvae and whether SB affects the commensal microbial compositions and functions. We also attempted to clarify the interaction between SB, commensal microbiota and host development by detecting the response of commensal microbiota after the intervention. The results show that SB significantly retarded the development of *D. melanogaster* larvae, shortened the life span, and changed the commensal microbial community. In addition, SB changed the transcription level of endocrine coding genes such as *ERR* and *DmJHAMT*. These results indicate that the slow down in *D. melanogaster* larvae developmental timing and shortened life span of adult flies caused by SB intake may result from the changes in endocrine hormone levels and commensal microbiota. This study provided experimental data that indicate SB could affect host growth and development of *D. melanogaster* through altering endocrine hormone levels and commensal microbial composition.

## Introduction

Sodium benzoate (SB) is the sodium salt of benzoic acid, represented by the chemical formula C_7_H_5_O_2_Na. It is widely used in the preservation of foods and drinks, such as jams, jellies, margarine, pickles, salads, sauces, vinegar, carbonated drinks, and fruit juices. SB is considered a “generally regarded as safe” compound by the United States Food and Drug Administration (FDA) (Food and Drug Administration [FDA], 2017). FDA has limited the amount of SB added to less than 0.1% (1,000 ppm) in food preservation (Lennerz et al., 2015). The World Health Organization’s official publication allows SB as an animal food additive for up to 0.1% (Lennerz et al., 2015). However, the increased consumption of processed and fast foods in modern society has led to excessive use of preservatives including SB and raised safety concerns for human physical health. The calls for the evaluation of SB safety and the potential harm of additive overuse are increasing.

The effects of SB on human and animal physical health are protective-toxic dual sides. SB was reported to be beneficial to many diseases. SB was highly beneficial for treating metabolic disorders, such as urea cycle disorders, and other diseases with hyperammonemia (Husson et al., 2016; NeSmith et al., 2016; Piper and Piper, 2017). Besides, SB was also found to be of potential therapeutic value for liver failure (De Las Heras et al., 2017), multiple sclerosis (Kundu et al., 2016), schizophrenia (Lane et al., 2013; Matsuura et al., 2015), early stage Alzheimer’s disease (Lin et al., 2014), Parkinson’s disease (Khasnavis and Pahan, 2012), and behavioral and psychological symptoms of dementia (Lin et al., 2020). However, Misel et al. (2013) showed that the significant high-dose dependence of SB may result in damage to the kidney, revealing the harmful effects of high-dose SB additions.

Many researchers have conducted experiments to test the toxicity of SB using animal models. SB was considered genotoxic, clastogenic, and neurotoxic, affecting cell cycle and DNA structure *via* intercalation (Linke et al., 2018). Mammals with chronic exposure to SB suffered from reduced food intake and growth (Nair, 2001). El-Shennawy et al. (2020) showed that SB significantly altered the reproductivity of male rats, as shown by the weight loss of reproductive organs, decreased sperm count and motility, and increased percentage of abnormal sperms. Consistent results were found in the study of Jewo et al. (2020), whereby the SB intake significantly reduced the total sperm count, and germ cell loss and sloughing of germinal epithelium were also found. Gaur et al. (2018) reported that SB changed developmental, morphological, biochemical, and behavioral features in developing zebrafish larva, including delayed hatching, pericardial edema, yolk sac edema, tail bending, oxidative stress, and anxiety-like behavior. In addition, SB induced liver histological alterations; increased lipid peroxidation and glutathione content; and declined catalase activity in the kidney tissues (Khodaei et al., 2019).

Gut microbiota is often referred to as a “superorganism” due to its vast number in the host body (Gill et al., 2006; Thursby and Juge, 2017). Gut microbiota draws dramatic attention in recent years owing to their extensive interactions with the host. The microbiota provides many beneficial effects on the health of the host, such as strengthening the gut integrity (Thursby and Juge, 2017), assisting the energy harvest (Murphy et al., 2010), protecting against pathogens (Bäumler and Sperandio, 2016), and regulating the host immunity (Gensollen et al., 2016). The antimicrobial effect has been well explored in the food and drink industries (Karabay et al., 2006; Zhao and Doyle, 2006). Stanojevic et al. (2009) found that the combination of sodium nitrite and SB synergistic inhibited 40% of *Escherichia coli*, *Staphylococcus aureus*, *Bacillus mucoides*, and *Candida albicans*. Nevertheless, the effect of SB on commensal gut microbial community compositions and functions of the host remains scarce.

*Drosophila melanogaster* (*D. melanogaster*) has the advantages of easy feeding, short life cycle, low maintenance cost, etc. In addition, it has conserved metabolism pathways with human. Therefore, it is considered as an ideal animal model in scientific studies. In this study, we investigated the effect of SB on the growth and development of *D. melanogaster* larvae, and whether SB affects the commensal microbial compositions and functions. We also aim to clarify the interaction between SB, commensal microbiota and host development by analyzing the response of commensal microbiota in flies fed with SB. The results show that 2,000 ppm of SB or higher significantly retarded the development of *D. melanogaster* larvae, shortened the life span, and altered the commensal microbial community.

## Materials and Methods

### *Drosophila melanogaster* Husbandry

The *D. melanogaster* stock used in this study was Canton-S-iso3A (Bloomington *Drosophila* Stock Center #9516, Indiana University, Bloomington, IN, United States). The flies were raised on a standard yeast-sucrose-cornmeal diet containing 25 g yeast, 40 g sucrose, 42.4 g maltose, 66.825 g cornmeal, 9.18 g soybean meal, 6 g agar, 0.5 g SB, 0.25 g nipagin, and 6.875 ml propionic acid per liter. The SB (Aladdin, AR, 99%) treatment media comprised a standard diet supplemented with SB at either 0, 200, 500, 1,000, 2,000, 3,000, or 5,000 ppm, respectively.

Mated females were transferred on an appropriate media for embryos laying, followed by transferring to SB treatment media. Fresh food was prepared every week and stored at 4°C to avoid desiccation. All flies were maintained under constant temperature (25°C) and humidity (65%) with a 12 h light-dark cycle.

### Developmental Timing Measurement

The larval-pupal and pupal-adult metamorphosis timing of individuals raised in different SB treatments was quantified by counting the number of pupae and adults emerging over time.

Adult weight was estimated using 1-day-old adults. For each condition, the weight of a pool of ten adult individuals was weighed using a precision balance [Mettler Toledo, MS105DU (Zurich, Switzerland)]. Each graph represents the mean of at least six biological replicates, including at least 10 individuals each.

### Survival Analysis

Two SB treatments (0 and 2,000 ppm) were selected to conduct survival analysis. Adult flies hatched from larvae growing on treatment media containing 0 and 2,000 ppm of SB were transferred to treatment media with 0 ppm and 2,000 ppm of SB and tested in the following four combinations, respectively. The group 0 + 0 means that survival analysis was tested on 0 ppm of SB media using adults hatched from larvae growing on 0 ppm of SB. The group 0 + 2000 means that survival analysis was tested on 2,000 ppm of SB media using adults hatched from larvae growing on 0 ppm of SB. The group 2000 + 0 means that survival analysis was tested on 0 ppm of SB media using adults hatched from larvae growing on 2,000 ppm of SB. The group 2000 + 2000 means that survival analysis was tested on 2,000 ppm of SB media using adults hatched from larvae growing on 2,000 ppm of SB.

Flies within 8 h of emergence were separated into single-sex groups (males and females) under light CO_2_ anesthesia. For both the male and female groups, 10 vials each containing 20 flies were used for the survival analysis of each treatment. Survival curves were determined by counting dead flies every 2–3 days, and the media was replaced every 5 days.

### 16S rRNA Gene Amplicon Analysis

#### Sample Collection and DNA Extraction

Adult flies (from both the SB fed and SB unfed groups) were collected and sequentially rinsed in 50% (v/v) bleach and 70% ethanol, after which they were washed extensively with phosphate buffer solution (PBS) before dissection. Guts from 15 flies in each sample were dissected in sterile PBS using sterile forceps, and the trachea, malpighian tubules, and crop were then carefully removed. Guts were collected in PBS on ice and then homogenized using a tissue grinder (Tiangen, OSE-Y20, Beijing, China) with a pestle (Tiangen, OSE-Y001, Beijing, China). Homogenized samples were stored at −80°C, and frozen gut samples were thawed at 37°C for 45 min in a 1.5 ml microcentrifuge tube and transferred to a 2 ml tube containing 600 μl LWA (BioBase, M2012-01, Chengdu, China). The supernatant was transferred to a 2 ml tube containing 30 μl BioBase Tissue beads (BioBase, M2012-01, Chengdu, China). After elution with WB and SPW buffers (BioBase, M2012-01, Chengdu, China), the beads were air-dried at room temperature. Finally, DNA was eluted using EB buffer (BioBase, M2012-01, Chengdu, China).

#### Illumina NovaSeq Sequencing and Bioinformatics Analysis

The 16S rRNA genes of V3–V4 regions were amplified using specific primers [341F (5′-CCTAYGGGRBGCASCAG-3′) and 806R (5′-GGACTACNNGGGTATCTAAT-3′)] with the barcode. PCR products were mixed in equidensity ratios, and then, the mixture of PCR products was purified with Qiagen Gel Extraction Kit (Qiagen, Germany).

Sequencing libraries were generated using TruSeq^®^ DNA PCR-Free Sample Preparation Kit (Illumina, United States) following the manufacturer’s recommendations, and index codes were added. The library quality was assessed using the Qubit^®^ 2.0 Fluorometer (Thermo Scientific, United States). At last, the qualified library was sequenced on an Illumina NovaSeq platform, and 250 bp paired-end reads were generated.

The bioinformatics analysis was conducted by following the “Atacama soil microbiome tutorial” of Qiime2 docs along with customized program scripts.^1^ De-multiplexed sequences from each sample were quality filtered and trimmed, de-noised, and merged, and then, the chimeric sequences were identified and removed using the QIIME2 dada2 plugin to obtain the feature table of amplicon sequence variant (ASV) (Callahan et al., 2016). The taxonomy table was generated by using the QIIME2 feature-classifier plugin aligning ASV sequences to a pretrained GREENGENES 13_8 99% database (Bokulich et al., 2018). Diversity metrics were calculated using the core-diversity plugin within QIIME2. Feature level alpha diversity indices, such as observed OTUs, Chao1 richness estimator, Shannon diversity index, and Simpson diversity index, were calculated to estimate the microbial diversity within an individual sample. Beta diversity distance measurements were performed to investigate the structural variation of microbial communities across samples and visualized *via* principal coordinate analysis (PCoA) (Vázquez-Baeza et al., 2013). Partial least squares discriminant analysis (PLS-DA) was introduced as a supervised model to reveal the commensal microbiota variation among groups using the “plsda” function in the R package “mixOmics” (Rohart et al., 2017). The potential KEGG Ortholog functional profiles of the commensal microbial communities were predicted with Phylogenetic Investigation of Communities by Reconstruction of Unobserved States (PICRUSt) (Langille et al., 2013). Linear discriminant analysis (LDA) effect size (LEfSe) analysis was conducted by the LEfSe module on the online galaxy website.

### Quantitative Reverse Transcription PCR to Assess Related Gene Expression of *Drosophila melanogaster*

Adult flies that emerged from SB media (0 and 2,000 ppm) within 8 h were selected. Whole bodies (15–20 flies per sample) were homogenized in the translation lookaside buffer (TLB) (BioBase, N1002-01) using a tissue grinder (Tiangen, OSE-Y20) with a pestle (Tiangen, OSE-Y001), and then, the total RNA was extracted following the user guidance of the RNA Extraction Kit (BioBase, N1002). RNA concentrations were measured using the Nanodrop 2000 Spectrophotometer (NanoDrop2000c; Thermo Scientific, Waltham, MA, United States), and 1 μg of total RNA per sample was reverse-transcribed using the FastKing First-Strand Synthesis System (Thermo, #K1641). Quantitative reverse transcription PCR (qRT-PCR) was performed using a Roche 480 II real-time PCR cycler (Roche, Basel, Switzerland) with 2 × Q3 QuantiNova SYBR Green II PCR Master Mix (TOLOBIO, 22204-1). The final mRNA expression fold change relative to the control was normalized to *rp49*. Primer sequences for qRT-PCR are shown in Supplementary Table 1.

### Statistical Analysis

Development timing, survival curves, and Mantel-Cox tests were analyzed by the GraphPad Prism 7 software (GraphPad Software, La Jolla, CA, United States). For other comparisons between two samples, two-tailed Student’s *t*-tests were used. For multiple comparisons, a one-way ANOVA with Tukey’s test was used. Graphs without special illustrations show the mean with error bars of 1 SD. Statistical significance is indicated by asterisks, where *p < 0.05, ^**^p < 0.01.

## Results

### Developmental Timing Measurement

**FIGURE 1:**
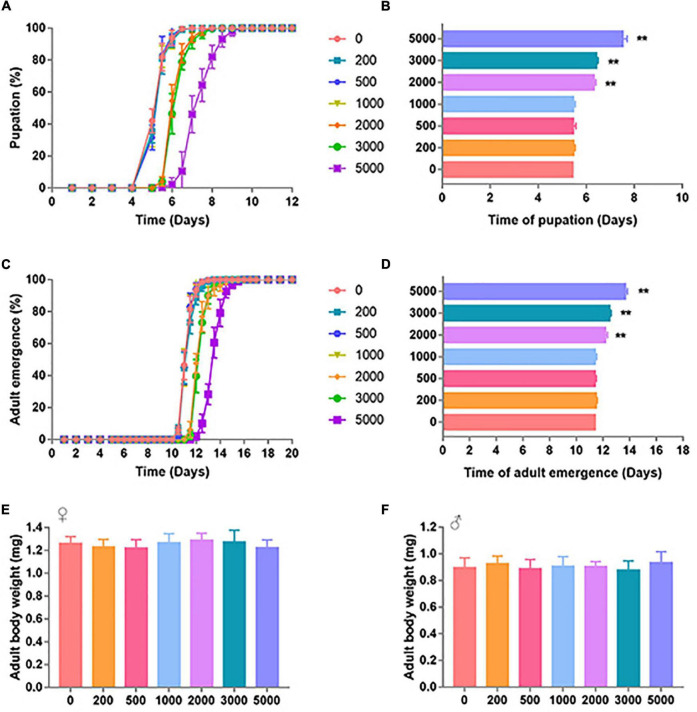
Development timing of SB-supplemented flies. **(A)** Pupation time of flies fed with SB. **(B)** Mean time of the pupation of flies fed with SB at 0, 200, 500, 1,000, 2,000, 3,000, and 5,000 ppm. **(C)** Adult emergence time of flies fed with SB. **(D)** Mean time of the adult emergence time of flies fed with 0, 200, 500, 1,000, 2,000, 3,000, and 5,000 ppm of SB. **(E)** Adult body weight of females fed with 0, 200, 500, 1,000, 2,000, 3,000, and 5,000 ppm of SB. **(F)** Adult body weight of males fed with 0, 200, 500, 1,000, 2,000, 3,000, and 5,000 ppm of SB. **p < 0.01.

The effect of SB on *D. melanogaster* development was characterized by pupation time, adult emergence time, and body weight. The developmental process of *D. melanogaster* was significantly retarded by high dosages of SB (Figure 1). Low dosages (0–1,000 ppm) of SB had no significant effect on the time of pupation (Figures 1A,B), whereas higher SB dosages (between 2,000 and 5,000 ppm) showed a longer time of pupation (Figures 1A,B). Consistent with the change of pupation time, low addition (0–1,000 ppm) of SB had no significant effect on the time of adult emergence (Figures 1C,D). SB addition between 2,000 and 5,000 ppm showed a longer time of adult emergence (Figures 1C,D). However, both male and female flies fed with SB showed no significant change in body weight (Figures 1E,F).

### Survival Analysis

**FIGURE 2:**
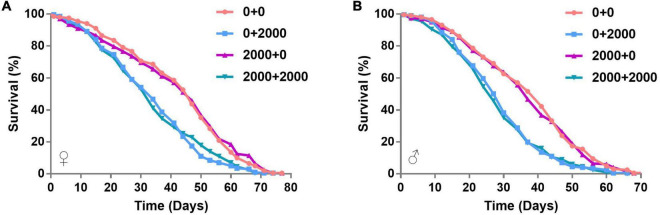
Survival curves of flies fed with SB. **(A)** Female files fed with 0, 200, 500, 1,000, 2,000, 3,000, and 5,000 ppm of SB. **(B)** Male files fed with 0, 200, 500, 1,000, 2,000, 3,000, and 5,000 ppm of SB.

To find out the effect of SB on the life span of adult flies, we further conducted the survival analysis. The groups of 0 + 0 and 2000 + 0 were set to explore whether early life (larvae)-only exposed to SB could affect adult life span. Mean survival rates of females were 52.88 and 52.22% in 0 + 0 and 2000 + 0 groups, respectively (*p* = 0.6184) (Table 1). Mean survival rates of males were 50.07 and 47.42% in 0 + 0 and 2000 + 0 groups, respectively (*p* = 0.6793) (Table 1). These results indicated that the treatment of SB in the larval stage-only did not affect adult life spans in both female and male groups (Figures 2A,B and Table 1). Similar results were found by comparing the mean life span of 0 + 2000 with 2000 + 2000 groups.

**TABLE 1:** Statistics for survival curves.

		Total no. of flies	Mean (% change)	Median (% change)	Log-rank
**Female**					
0 + 0	0 + 2000	200	52.88	60.95	*p* < 0.0001
	2000 + 0				*p* = 0.6184
	2000 + 2000				*p* < 0.0001
0 + 2000	2000 + 0	200	44.13 (−8.75%)	38.81 (−22.14%)	*p* < 0.0001
	2000 + 2000				*p* = 0.7802
2000 + 0	2000 + 2000	200	52.22 (−0.66%)	59.20 (−1.74%)	*p* < 0.0001
2000 + 2000		200	45.96 (−6.92%)	38.06 (−22.87%)	−
**Male**					
0 + 0	0 + 2000	200	50.27	54.48	*p* < 0.0001
	2000 + 0				*p* = 0.6793
	2000 + 2000				*p* < 0.0001
0 + 2000	2000 + 0	200	43.84 (−6.42%)	33.58 (−20.90%)	*p* < 0.0001
	2000 + 2000				*p* = 0.6220
2000 + 0	2000 + 2000	200	47.42 (−2.85%)	46.77 (−7.71%)	*p* < 0.0001
2000 + 2000		200	44.49 (−5.77%)	35.32 (−19.15%)	−

*Cohort sizes, mean and median life spans, percentage changes, and log-rank (Mantel-Cox) tests for survival curves in this study.*

The groups of 0 + 0 and 0 + 2000 were set to explore whether continuous adult exposure to SB could affect the adult life span. Mean survival rates of females were 52.88 and 44.13% in 0 + 0 and 0 + 2000 groups, respectively (*p* < 0.0001) (Table 1). Mean survival rates of males were 50.07 and 43.84% in 0 + 0 and 0 + 2000 groups, respectively (*p* < 0.0001) (Table 1). These results demonstrate that the treatment of SB in adult stage-only significantly shortened the life spans in both female and male groups (Figures 2A,B and Table 1). Similar results were found by comparing the mean life span of 2000 + 0 with 2000 + 2000 groups.

The above results indicate that SB treated in the larval stage-only would not change the life span, whereas SB treated in the adult stage would shorten the life span.

### Commensal Microbiota

#### Sodium Benzoate Changed the Commensal Microbial Diversity of Flies

To investigate the effect of SB on commensal microbiota, we further conducted 16S rRNA gene amplicon sequencing. A total of 1,606,655 high-quality sequences were generated *via* sequencing, and a 99% identity cutoff was used to define each OTU by QIIME2 dada2. The OTU numbers ranged from 789 to 1,122 for each group. The 16S sequence data generated in this study were submitted to the NCBI SRA database (accession number PRJNA774185).

A large number abundance of *Wolbachia* was found in the sequencing data. This indicated that some *Wolbachia* contamination may occur. *Wolbachia* is an intracellular endosymbiont mostly found in the reproductive tract of *Drosophila*. Thus, to avoid the effect of *Wolbachia* and to get a more accurate effect of SB on the commensal gut microbiota of *Drosophila*, we conducted the microbial analysis based on the *Wolbachia*-excluded data in the “Results” section (Figures 4–7 and Tables 2, 3). In addition, to draw more accurate conclusions, the analysis of *Wolbachia*-included data was also carried out (Supplementary Figures 1–4 and Supplementary Tables 2, 3).

**TABLE 2:** PERMANOVA of microbiota based on Bray–Curtis distance of *Wolbachia*-excluded data.

Group 1	Group 2	Sample size	Permutations	pseudo-F	*P*-value
SB0 ppm	SB2000 ppm	16	999	12.117	0.002
SB0 ppm	SB5000 ppm	16	999	14.759	0.001
SB2000 ppm	SB5000 ppm	16	999	1.205	0.323

**TABLE 3:** PERMANOVA of microbial function based on Bray–Curtis distance of *Wolbachia*-excluded data.

Group 1	Group 2	Sample size	Permutations	pseudo-F	*P*-value
SB0 ppm	SB2000 ppm	16	999	72.177	0.001
SB0 ppm	SB5000 ppm	16	999	32.819	0.001
SB2000 ppm	SB5000 ppm	16	999	2.5958	0.12

**FIGURE 3:**
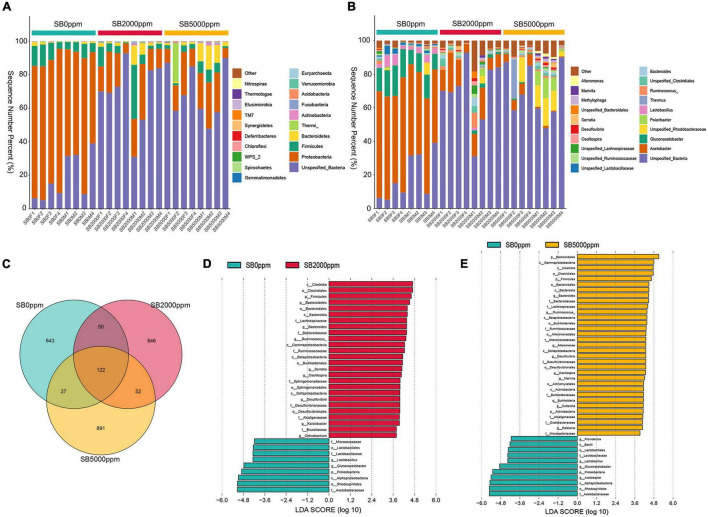
Commensal microbial composition and species enrichment analysis of *Wolbachia*-excluded data. **(A)** Commensal microbial composition at the phylum level. **(B)** Commensal microbial composition at the genus level. **(C)** Common and unique OTUs number analysis. **(D)** Linear discriminant analysis (LDA) scores of commensal microbial species in flies fed with 2,000 ppm of SB. **(E)** LDA scores of commensal microbial species in flies fed with 5,000 ppm of SB.

The commensal microbiota of phylum level consisted of Proteobacteria, Firmicutes, Bacteroidetes, and Actinobacteria, which were consistent with the *Wolbachia*-included data (Figure 3A and Supplementary Figure 1A). Genus levels mainly included *Acetobacter*, *Gluconacetobacter*, and *Lactobacillus*, which were also consistent with the *Wolbachia*-included data (Figure 3B and Supplementary Figure 1B). There were 122 shared OTUs between 0, 2,000, and 5,000 ppm of SB groups (Figure 3C). There were 643, 846, and 891 unique OTUs in 0, 2,000, and 5,000 ppm of SB groups, respectively (Figure 3C). Species enrichment analysis showed that the dominant commensal bacteria of Acetobacteraceae, *Gluconacetobacter*, and *Lactobacillus* were lacking in flies fed with 2,000 ppm of SB (Figure 3D), and *Acetobacter* was further depleted in flies fed with 5,000 ppm of SB (Figure 3E). In *Wolbachia*-included data, *Acetobacter* was depleted both in flies fed with 2,000 and 5,000 ppm of SB (Supplementary Figure 1D).

**FIGURE 4:**
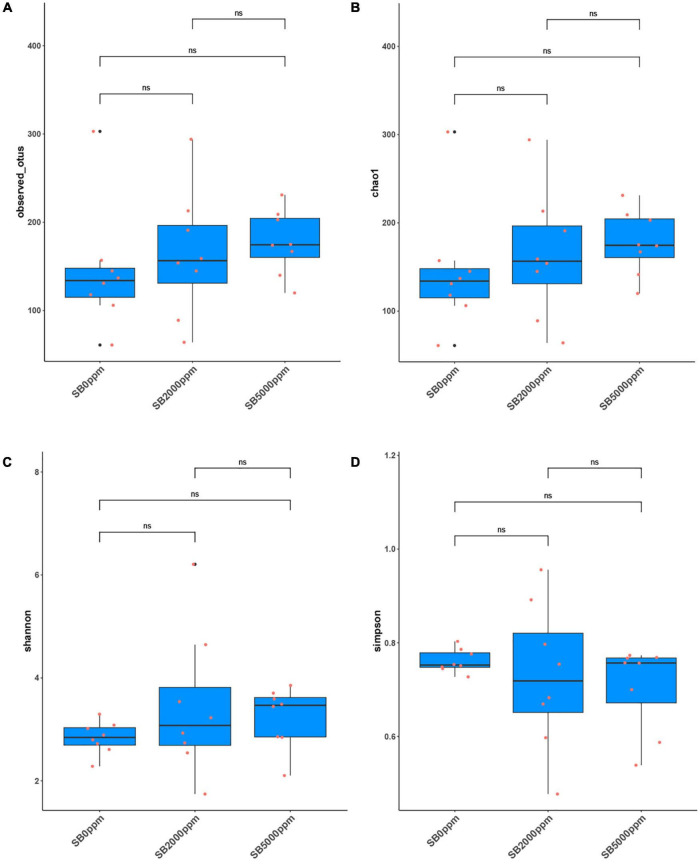
Alpha diversity of flies’ commensal microbiota fed with SB at the genus level of *Wolbachia*-excluded data. **(A)** Observed OTUs. **(B)** Chao1. **(C)** Shannon. **(D)** Simpson.

**FIGURE 5:**
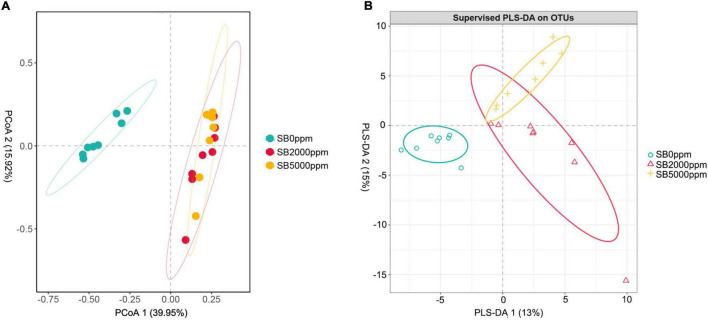
Beta diversity of flies’ commensal microbiota fed with SB at genus level of *Wolbachia*-excluded data. **(A)** Principal coordinate analysis (PCoA) score plot based on Bray-Curtis distance. **(B)** Partial least squares discriminant analysis (PLS-DA) plot.

**FIGURE 6:**
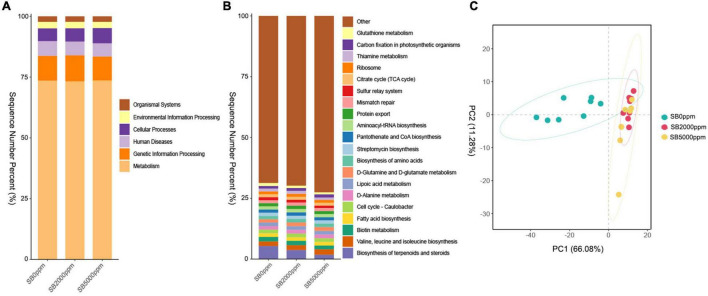
Function prediction of commensal microbiota of *Wolbachia*-excluded data. **(A)** KEGG pathway at L1 level. **(B)** KEGG pathway at L3 level. **(C)** Principal component analysis (PCA) of commensal microbial function at L3 level.

**FIGURE 7:**
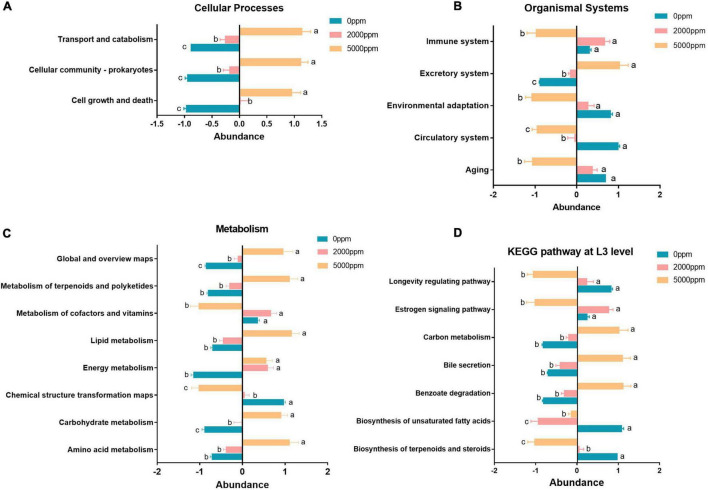
Significant pathway (ANOVA) of the commensal microbial function of *Wolbachia*-excluded data. **(A)** Cellular processes. **(B)** Organismal systems. **(C)** Metabolism. **(D)** KEGG pathway at L3 level. The same letters (a, b, c) next to the bars indicate that there is a significant difference between the two groups and, otherwise, no significant difference between the two groups.

The analysis of observed OTUs showed no significance in the sequencing depth index between SB supplemented flies and no SB supplemented flies whether *Wolbachia* was excluded or not (Figure 4A and Supplementary Figure 2A). Chao1 analysis showed that commensal microbiota richness also had no significance between SB supplemented flies and no SB supplemented flies whether *Wolbachia* was excluded or not (Figure 4B and Supplementary Figure 2B). However, it was uncertain that the commensal microbial diversity was affected by SB due to the inconsistent results of the Shannon and Simpson index between the *Wolbachia*-excluded data (Figures 4C,D) and the *Wolbachia*-included data (Supplementary Figures 2C,D).

Commensal microbiota of flies fed with SB was well separated from that of flies fed with no SB whether *Wolbachia* was excluded or not (Figures 5A,B and Supplementary Figures 3A,B). Results of PERMANOVA also manifested that the commensal microbiota of SB-fed flies was significantly different from that of no SB-fed flies (*p* = 0.002, 0.001, Table 2, *p* = 0.001, 0.002, Supplementary Table 2). These results indicated that the addition of SB changed the commensal microbiota of *D. melanogaster* significantly.

#### Sodium Benzoate Changed the Commensal Microbial Function of Flies

To further clarify whether the commensal microbial function is modified in a similar manner to shift along with the commensal microbial composition, we conducted function prediction by PICRUSt. The function of KEGG pathways includes metabolism, genetic information processing, human diseases, cellular processes, environmental information processing, and organismal systems at the L1 level in all groups (Figure 6A). KEGG pathway at the L3 level mainly included biosynthesis of terpenoids and steroids; valine, leucine, and isoleucine biosynthesis; biotin metabolism; fatty acid biosynthesis; cell cycle-caulobacter; D-alanine metabolism; lipoic acid metabolism; D-glutamine and D-glutamate metabolism; biosynthesis of amino acids; streptomycin biosynthesis; pantothenate and CoA biosynthesis; aminoacyl-tRNA biosynthesis; protein export; mismatch repair; sulfur relay system; citrate cycle (TCA cycle); ribosome; thiamine metabolism, carbon fixation in photosynthetic organisms; and glutathione metabolism in all groups (Figure 6B). For *Wolbachia*-included data, similar results were found in commensal microbial function at the KEGG L1 level; however, there are some inconsistencies in commensal microbial function at the KEGG L3 level (Supplementary Figures 4A,B). The results of PCA and PERMANOVA showed that the commensal microbial function of flies fed with SB was significantly different from that of flies fed with no SB whether *Wolbachia* was excluded or not (Figure 6C, Table 3, Supplementary Figure 4C, and Supplementary Table 3).

The results of KEGG pathway prediction manifest that cell growth and death, cellular community–prokaryotes, transport, and catabolism pathway abundance were reduced by SB (Figure 7A). Aging, circulatory system, environmental adaptation, and immune system were reduced by SB at 5,000 ppm, whereas the excretory system was increased by SB (Figure 7B). Nucleotide metabolism, carbohydrate metabolism, energy metabolism, lipid metabolism, metabolism of terpenoids and polyketides, and global and overview maps were increased by SB at 5,000 ppm; however, chemical structure transformation and metabolism of cofactors and vitamins were reduced by SB at 5,000 ppm (Figure 7C). For KEGG L3 level, benzoate degradation, bile secretion, and carbon metabolism pathway abundance were increased by SB at 5,000 ppm, while biosynthesis of terpenoids and steroids, biosynthesis of unsaturated fatty acids, estrogen signaling pathway, and longevity regulating pathway abundance were reduced by SB at 5,000 ppm (Figure 7D).

### Gene Expressions of Males and Females Fed With Sodium Benzoate

**FIGURE 8:**
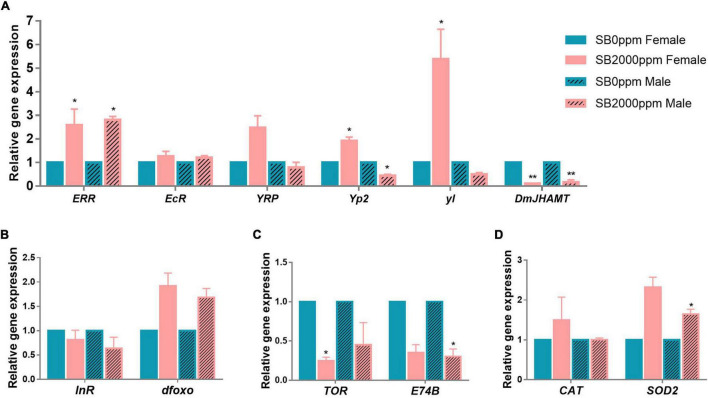
Gene expressions of flies fed with SB. **(A)** Hormone-related genes. **(B)** IIS pathway-related genes. **(C)** TOR pathway-related genes. **(D)** Antioxidant enzymes-related genes. The significance was achieved by comparing SB with the control groups. *p < 0.05, **p < 0.01.

To explore mechanisms of the retarded development of *D. melanogaster* by SB, we tested the expressions of hormone-, insulin/insulin-like growth factor (IGF-1) signaling (IIS) pathway-, the target of rapamycin (TOR) pathway-, and antioxidant enzymes-related genes. The expression of *ERR* was increased in both female and male flies (Figure 8A). *EcR* and *YPR* were not affected by SB (Figure 8A). *DmJHAMT* expression was reduced by SB in both female and male flies (Figure 8A). The *yl* was highly expressed in females (Figure 8A); *Yp2* expression was also increased in females, while it decreased in males (Figure 8A). It was shown that the expressions of *InR* and *dfoxo* of the IIS pathway were not altered in both male and female flies fed with SB (Figure 8B). *TOR* and *E74B* gene expressions were reduced in females and males, respectively (Figure 8C). For antioxidant enzyme-related genes, *CAT* was not changed by SB, and *SOD2* was increased in male flies (Figure 8D).

## Discussion

In this study, we attempted to explore the effect of SB on host development and the host commensal microbial community using *D. melanogaster*. Our results demonstrate that 2,000 ppm of SB or higher significantly slowed down the larvae development and shortened the adult life span of *D. melanogaster*. These results indicated that SB is harmful to host physical health when SB concentrations are added to food at 2,000 ppm or higher. This is consistent with the results of previous studies that showed high concentrations of SB would be harmful to the health, as evidenced by reduced reproductivity, developmental defects, oxidative stress, and anxiety-like behavior (Gaur et al., 2018; Khodaei et al., 2019; El-Shennawy et al., 2020; Jewo et al., 2020). Interestingly, the no significant difference in survival rates between 0 + 0 and 2000 + 0 groups indicates that the exposure of SB to flies in early life-only may not affect the life span (Figures 2A,B). However, the significant difference in the survival rates between 0 + 0 and 0 + 2000 groups indicates that continuous adult exposure had more harmful effects on *Drosophila* life span than early life-only exposure (Figures 2A,B).

Of note, the commensal microbial composition was changed in *D. melanogaster* fed with 2,000 or 5,000 ppm of SB. The symbionts could modulate the life-history traits of *D. melanogaster*, including juvenile growth, life span, and behavior (Erkosar et al., 2013; Lee and Brey, 2013; Strigini and Leulier, 2016). An increase in the unique OTUs after SB intake was found in this study. This could be explained by the decrease in the dominant species resulting in the rearrangement of the commensal microbiota ecological niche in *D. melanogaster*, and then, the rearrangement of the commensal microbiota ecological niche led to the increased abundance of unique OTUs.

Acetobacteraceae and Lactobacillaceae are two dominant families of *Drosophila* commensal intestinal tract bacteria in both wild and laboratory stains (Erkosar et al., 2013; Lee and Brey, 2013; Storelli et al., 2018), playing important roles in the development of *D. melanogaster*, including the larval growth, fecundity, immunity, and life cycle (Shin et al., 2011; Sansone et al., 2015; Elgart et al., 2016). *Lactobacillus plantarum* promoted the systemic growth of *D. melanogaster* by modulating hormonal signals and TOR-dependent nutrient sensing (Storelli et al., 2011), induced the intestinal peptidases transcription, and increased the amino acid uptake of *D. melanogaster* larvae (Storelli et al., 2018). Téfit and Leulier (2017) demonstrated that *Lactobacillus plantarum* promotes the growth of *Drosophila* larvae and leads to earlier metamorphosis and adult emergence upon nutrient scarcity compared with axenic individuals. In this study, SB intake significantly reduced the abundance of *Lactobacillus*, which may be one of the factors that result in delaying of the *D. melanogaster* larval development. Nevertheless, inconsistent results were found in the effect of *Lactobacillus* on the life span of *Drosophila*. Téfit and Leulier (2017) observed a life span extension in nutritionally challenged males, while Fast et al. (2018) displayed that mono-association of adult *Drosophila* with *Lactobacillus plantarum* curtailed adult longevity compared with germ-free flies. Thus, in this study, it is uncertain whether the shortened life span is related to the reduced *Lactobacillus* or not. *Acetobacter* could promote the growth and reproduction of *Drosophila* host (Shin et al., 2011; Fridmann-Sirkis et al., 2014; Elgart et al., 2016). *Acetobacter* increased triglycerides and starvation resistance, increased fecundity, enhanced larval growth, and shortened the longevity in *D. melanogaster*, whereas co-evolution confers that the host more fits to the adverse environment (Obata et al., 2018). We also observed a decreased abundance in *Acetobacter* by SB. We further speculated that the corresponding functional loss associated with *Acetobacter* may also be one of the reasons for postponing the development of *D. melanogaster* larvae.

The function of commensal microbiota in flies fed with 2,000 or 5,000 ppm of SB significantly differed from that in flies fed with no SB (Figure 6C, Table 3, Supplementary 4C, Supplementary Table 3). Pathways of metabolism, genetic information processing, human diseases, cellular processes, environmental information processing, and organismal systems were significantly changed by SB at concentrations of 2,000 ppm or higher. Moreover, we also found metabolism pathways at L3 level significantly changed by SB, including an increased abundance of benzoate degradation, bile secretion, and carbon metabolism pathways and a reduced abundance of biosynthesis of terpenoids and steroids, biosynthesis of unsaturated fatty acids, estrogen signaling pathway, and longevity regulating pathways (Figure 7D). The increased abundance of benzoate degradation may result from the high concentrations of SB intake. The reduced biosynthesis of terpenoids and steroids and estrogen signaling pathway may relate to the changed endocrine system by SB intake. Unsaturated fatty acids have effects on blood lipid concentrations, blood pressure, inflammatory response, arrhythmia and endothelial function (Lunn and Theobald, 2006). Unsaturated fatty acids also play a role in antimicrobial activity (Zheng et al., 2005). The decreased biosynthesis of unsaturated fatty acids may lead to a reduced protection of the host. The decreased abundance of longevity regulating pathway may explain a bit of the shortened life span by SB at concentrations of 2,000 ppm or higher. Strangely, the above pathways were changed more significantly in flies fed with 5,000 ppm of SB than in flies fed with 2,000 ppm. However, the physiological changes already occurred in flies fed with 2,000 ppm of SB. The reason needs to be explored further.

In addition, some contamination of *Wolbachia* was found in this study. This may result from incomplete separation of the reproductive tract or the contamination during the reproductive tract dissection. We further compared the analysis results of *Wolbachia*-included data with *Wolbachia*-excluded data. Except for the further depletion of *Acetobacter* by 5,000 ppm of SB, similar results were found in differential species and commensal microbial composition whether the *Wolbachia* was excluded or not. Besides, the commensal microbial function was significantly affected by SB whether the *Wolbachia* was excluded or not. *Wolbachia* was a common symbiont with insects, playing important roles in modifications of host fitness. *Wolbachia* infection increased the reproduction and produced a positive- or a non-effect on host survival in *D. melanogaster* (Fry and Rand, 2002; Fry et al., 2004; Gruntenko et al., 2017). However, it is uncertain that the retarded development of larvae and shortened life span of adult flies were related to *Wolbachia*. Therefore, further studies performed in *Wolbachia*-free *D. melanogaster* are needed to exclude the effect of *Wolbachia*.

Larval development was reported to be most related to the endocrine hormone. Juvenile hormones (JHs), a family of sesquiterpenoid hormones, are a key endocrine regulator of insects’ metamorphosis, development, growth, reproduction, and aging (Gilbert et al., 2000; Meiselman et al., 2017). JH regulates insect metamorphosis, including preventing immature larvae from going through precocious larval-pupal transition and increasing the number of molts (Kayukawa et al., 2017). JH acid O-methyltransferase (JHAMT) is the enzyme that transfers a methyl group from S-adenosyl-L-methionine (SAM) to the carboxyl group of JH acids, resulting in the catalyzation of the final step of the JH to produce active JHs in Lepidoptera (Shinoda and Itoyama, 2003; Niwa et al., 2008). The decrease in *DmJHAMT* transcription of flies fed with SB indicates that the SB may have delayed the insect molting, leading to the slow development of *Drosophila* larvae.

Ecdysone receptor (EcR) is a nuclear hormone receptor that activates the arthropod steroid hormones ecdysteroids and regulates molting, metamorphosis, reproduction, diapause, and innate immunity in insects (Koelle et al., 1991; Yao et al., 1992; Thomas et al., 1993). EcR plays a considerable role in the larval-to-prepupal transition of *Drosophila* (Uyehara and McKay, 2019). EcR isoforms are required for larval molting and neuron remodeling during metamorphosis in insects (Schubiger et al., 1998; Xu et al., 2020). In addition, many studies have shown that EcR is a key factor affecting life span and reproduction (Antoniewski et al., 1996; Thummel, 1996; Riddiford et al., 2000). Reduced EcR levels lead to increased longevity and stress resistance in adults (Simon et al., 2003; Tricoire et al., 2009). In this study, the mRNA level of *EcR* remained unchanged in flies fed with SB, suggesting that the retarded larval development and shortened life span by SB were not likely through the *EcR* regulation.

Estrogen-related receptor (ERR), another nuclear hormone receptor, is a critical metabolic transition during *Drosophila* development. *dERR* is involved in a transcriptional switch during pupal development that determines the adult fly’s glucose oxidation and lipogenesis (Beebe et al., 2020). It also reported that *dERR* is essential for carbohydrate metabolism in larval stages, and *dERR* mutants die as larvae (Tennessen et al., 2011). Our study found that *ERR* transcripts increased in flies fed with SB. Nevertheless, the relationship between the retarded development of flies resulting from SB and the increased *ERR* transcripts still needs to be investigated.

The yolk protein (YP) receptor (YPR) is a receptor of egg yolk protein in *D. melanogaster*. Vitellogenin as a precursor of egg yolk protein has become a well-established biomarker for measuring the effect of environmental chemicals on estrogenic activity (Tufail and Takeda, 2009). We have observed no significant changes in the *YPR* level. *Yp2* was significantly increased in females fed with 2,000 ppm of SB, whereas it decreased in males. Yolkless (*yl*) gene was significantly enhanced in females fed with 2,000 ppm of SB. Regarding the inconsistency in the changes of *Yp2* and *yl* expression between males and females, it is uncertain that *Yp2* and *yl* were related to the retarded development of larvae in this study.

The IIS pathway could be another factor that may affect *Drosophila* development. A previous study has demonstrated that IIS pathway controls the formation of larva, stress-resistant and long-lived (Kapahi et al., 2004). IIS directly regulates *Drosophila* developmental transition timing through the production of the molting hormone ecdysone (Ghosh, 2021). A previous study found that an increased IIS improved the larval growth rate and promoted the metamorphosis of *Drosophila*, which was accompanied by the synthesis of precocious ecdysone and increased transcription of ecdysone biosynthetic genes (Walkiewicz and Stern, 2009). For example, Tu et al. (2005) showed that IIS pathway may regulate JH synthesis through the control of JH regulatory neuropeptides. Moreover, it was reported that there is a feedback loop in the interaction of IIS and JH, where IIS controlled stress resistance through JH/dopamine signaling regulation (Gruntenko and Rauschenbach, 2018). There is also an interaction between the commensal microbiota and IIS. Increased abundance of *Wolbachia* could enhance IIS through an evolutionary explanation (Ikeya et al., 2009). However, *InR* and *dfoxo*, as two key factors of IIS, remained unchanged by 2,000 ppm of SB in our study. This indicated that the altered developmental rate of *Drosophila* was not through IIS pathway.

The TOR pathway has emerged as the major regulator of growth and size in *Drosophila* (Kapahi et al., 2004). TOR pathway is a highly conserved nutrient-sensing pathway that regulates growth, metabolism, and aging (Bjedov et al., 2010). *L. plantarum* association impacts both InR and Ecd signaling in larvae, and *L. plantarum* exerts its benefit by acting genetically upstream of the TOR-dependent host nutrient sensing system controlling hormonal growth signaling (Storelli et al., 2011). Our study shows that *TOR* decreased only in females and *E74B* decreased only in males fed with 2,000 ppm of SB, which means that SB may decrease the TOR pathway to some extent.

Overall, we investigated the effect of SB on the growth and development of *D. melanogaster* larvae and whether SB affects the commensal microbial compositions and functions. Our results will help to clarify the interaction between SB, commensal microbiota, and host development. In conclusion, we have found a longer larval–pupal and pupal–adult metamorphosis timing and a shorter life span in flies fed with 2,000 ppm of SB or higher. Transcripts of endocrine encoding genes including *ERR* and *DmJHAMT* were changed. The commensal microbial compositions and functions were also found to be changed in adult flies fed with 2,000 ppm of SB or higher. These results indicate that the retarded *Drosophila* larvae developmental time and shortened life span by SB may be caused by the changes in endocrine level and commensal microbiota. Further multi-omics studies, such as metagenomics, metabolomics, and transcriptomics, are needed to verify this mechanism.

## Data Availability Statement

The 16S sequence data generated in this study were submitted to the NCBI SRA database, accession number PRJNA774185 (https://www.ncbi.nlm.nih.gov/search/all/?term=PRJNA774185).

## Ethics Statement

Ethical review and approval was not required for the animal study because the manuscript presents results of research on invertebrate animals *(Drosophila melanogaster)*.

## Author Contributions

YD and QP contributed to the conception and design of the study. YD, ZD, LS, and DZ performed the experiments. YD and DZ collected and analyzed the data. YD wrote the manuscript. CX, SZ, LF, and HL reviewed and polished the manuscript. All authors contributed to the manuscript revision, read, and approved the submitted version.

## Conflict of Interest

The authors declare that the research was conducted in the absence of any commercial or financial relationships that could be construed as a potential conflict of interest.

## Publisher’s Note

All claims expressed in this article are solely those of the authors and do not necessarily represent those of their affiliated organizations, or those of the publisher, the editors and the reviewers. Any product that may be evaluated in this article, or claim that may be made by its manufacturer, is not guaranteed or endorsed by the publisher.

## Footnotes

1
https://docs.qiime2.org/2019.1/

## Supplementary Material

Supplementary Figure 1 Commensal microbial composition and species enrichment analysis of *Wolbachia*-included data. **(A)** Commensal microbial composition at the phylum level. **(B)** Commensal microbial composition at the genus level. **(C)** Common and unique OUTs number analysis. **(D)** Linear discriminant analysis (LDA) scores of commensal microbial species in flies fed with 2,000 ppm of SB. **(E)** LDA scores of commensal microbial species in flies fed with 5,000 ppm of SB.

Supplementary Figure 2 Alpha diversity of flies’ commensal microbiota of *Wolbachia*-included data fed with SB at the genus level. **(A)** Observed OTUs. **(B)** Chao1. **(C)** Shannon. **(D)** Simpson.

Supplementary Figure 3 Beta diversity of flies’ commensal microbiota of *Wolbachia*-included data fed with SB at the genus level. **(A)** Principal coordinate analysis (PCoA) score plot based on Bray-Curtis distance. **(B)** Partial least squares discriminant analysis (PLS-DA) plot.

Supplementary Figure 4 Function prediction of commensal microbiota of *Wolbachia*-included data. **(A)** KEGG pathway at L1 level. **(B)** KEGG pathway at L3 level. **(C)** Principal component analysis (PCA) of commensal microbial function at L3 level.
