# Supplementary material for: Sodium Benzoate Delays the Development of Drosophila melanogaster Larvae and Alters Commensal Microbiota in Adult Flies
Source: Front Microbiol. 2022 Jun 22;13:911928. doi: 10.3389/fmicb.2022.911928 (PMC9257017; doi:10.3389/fmicb.2022.911928)
Supplement: Supplementary file 1 [file Table_1.DOCX]

**Supplementary Table 1. Primer Sequences of qRT-PCR used in this study**

| Gene | Primer sequences (5'→3') | Reference |
| --- | --- | --- |
| *ERR* | CCTGAAATCCTCGCCCTC; CCATTGCCGCCACTTGTA | ([Liu, Li, Zhao, Zhang, & Gu, 2014](#_ENREF_3)) |
| *EcR* | AAGGAAGGTATATTGCGCGC; GGAGAACCAATGTGCGATGA | ([Liu et al., 2014](#_ENREF_3)) |
| *YPR* | AGCAGCCATGCGCTCAGCTA; GCATTACGGGTTGTTCGCTCAGG | ([Liu et al., 2014](#_ENREF_3)) |
| *Yp2* | GGCGCTACGCTGTTGGA; GGGCTTGAAGACATGGTTGAA | ([Bovier, Rossi, Mita, & Digilio, 2018](#_ENREF_1)) |
| *yl* | CGAACGAGGATGCTTAAATCTATGT; CGGTTCCGGCATAGGAATT | ([Bovier et al., 2018](#_ENREF_1)) |
| *DmJHAMT* | AAACATATGAATCAGGCCTCTCTATATCAG; AACTCGAGGACTCTGTTAACAAATGCAATTACTG | ([Niwa et al., 2008](#_ENREF_4)) |
| *InR* | AACAGTGGCGGATTCGGTT; TACTCGGAGCATTGGAGGCAT | ([Obata, Fons, & Gould, 2018](#_ENREF_5)) |
| *dfoxo* | TCGAGTGCAATGTCGAGGAG; AGCGGTATATTGATGTCCAGCAG | ([Boyd et al., 2011](#_ENREF_2)) |
| *Tor* | GCTCAGAGGCGAGAGACAAG; CCAGCTCACGGAGGATAAAG | ([Boyd et al., 2011](#_ENREF_2)) |
| *E74B* | GAATCCGTAGCCTCCGACTGT; AGGAGGGAGAGTGGTGGTGTT | ([Obata et al., 2018](#_ENREF_5)) |
| *cat* | CCTCTGATTCCTGTGGGCAA; GACGACCATGCAGCATCTTG | ([Staats et al., 2018](#_ENREF_6)) |
| *sod2* | AATTTCGCAAACTGCAAGC; TGATGCAGCTCCATGATCTC | ([Obata et al., 2018](#_ENREF_5)) |
| *Rp49* | CAGTCGGATCGATATGCTAAGC; GGCATCAGATACTGTCCCTTGAA | ([Bovier et al., 2018](#_ENREF_1)) |

**References**

Bovier, T. F., Rossi, S., Mita, D. G., & Digilio, F. A. (2018). Effects of the synthetic estrogen 17-α-ethinylestradiol on *Drosophila melanogaster*: Dose and gender dependence. *Ecotoxicology and Environmental Safety, 162*, 625-632.

Boyd, O., Weng, P., Sun, X., Alberico, T., Laslo, M., Obenland, D. M., Zou, S. (2011). Nectarine promotes longevity in *Drosophila melanogaster*. *Free Radical Biology and Medicine, 50*(11), 1669-1678.

Liu, T., Li, Y., Zhao, X., Zhang, M., & Gu, W. (2014). Ethylparaben affects lifespan, fecundity, and the expression levels of *ERR*, *EcR* and *YPR* in *Drosophila melanogaster*. *Journal of Insect Physiology, 71*, 1-7.

Niwa, R., Niimi, T., Honda, N., Yoshiyama, M., Itoyama, K., Kataoka, H., & Shinoda, T. (2008). Juvenile hormone acid O-methyltransferase in *Drosophila melanogaster*. *Insect Biochemistry and Molecular Biology, 38*(7), 714-720.

Obata, F., Fons, C. O., & Gould, A. P. (2018). Early-life exposure to low-dose oxidants can increase longevity via microbiome remodelling in *Drosophila*. *Nature Communications, 9*(1), 975.

Staats, S., Wagner, A., Kowalewski, B., Rieck, F., Soukup, S., Kulling, S., & Rimbach, G. (2018). Dietary resveratrol does not affect life span, body composition, stress response, and longevity-related gene expression in *Drosophila melanogaster*. *International Journal of Molecular Sciences, 19*(1), 223.

**Supplementary Table 2.** PERMANOVA of microbiota based on bray-curtis distance of *Wolbachia*-included data.

| Group 1 | Group 2 | Sample size | Permutations | pseudo-F | *p*-value |
| --- | --- | --- | --- | --- | --- |
| SB0ppm | SB2000ppm | 16 | 999 | 15.00842 | 0.001 |
| SB0ppm | SB5000ppm | 16 | 999 | 17.7202 | 0.002 |
| SB2000ppm | SB5000ppm | 16 | 999 | 1.45644 | 0.201 |

**Supplementary Table 3.** PERMANOVA of microbial function based on bray-curtis distance of *Wolbachia*-included data.

| Group 1 | Group 2 | Sample size | Permutations | pseudo-F | *p*-value |
| --- | --- | --- | --- | --- | --- |
| SB0ppm | SB2000ppm | 16 | 999 | 9.6781 | 0.003 |
| SB0ppm | SB5000ppm | 16 | 999 | 10.646 | 0.001 |
| SB2000ppm | SB5000ppm | 16 | 999 | 0.67227 | 0.477 |
