# Supplementary figures and images for: Sodium Benzoate Delays the Development of Drosophila melanogaster Larvae and Alters Commensal Microbiota in Adult Flies
Source: Front Microbiol. 2022 Jun 22;13:911928. doi: 10.3389/fmicb.2022.911928 (PMC9257017; doi:10.3389/fmicb.2022.911928)

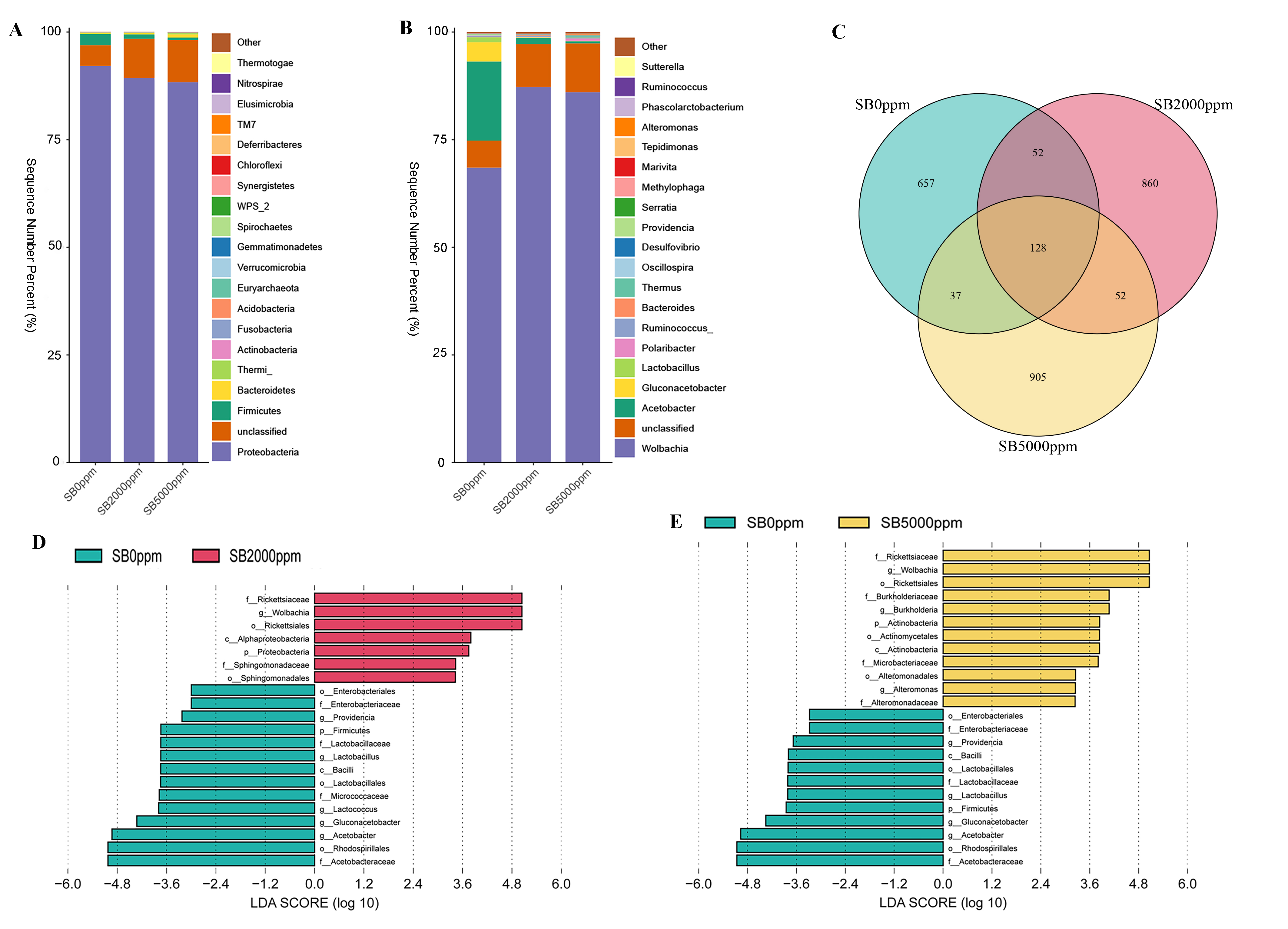

Supplement: Supplementary Figure 1 — Commensal microbial composition and species enrichment analysis of Wolbachia-included data. (A) Commensal microbial composition at the phylum level. (B) Commensal microbial composition at the genus level. (C) Common and unique OUTs number analysis. (D) Linear discriminant analysis (LDA) scores of commensal microbial species in flies fed with 2,000 ppm of SB. (E) LDA scores of commensal microbial species in flies fed with 5,000 ppm of SB. [file Image_1.JPEG]

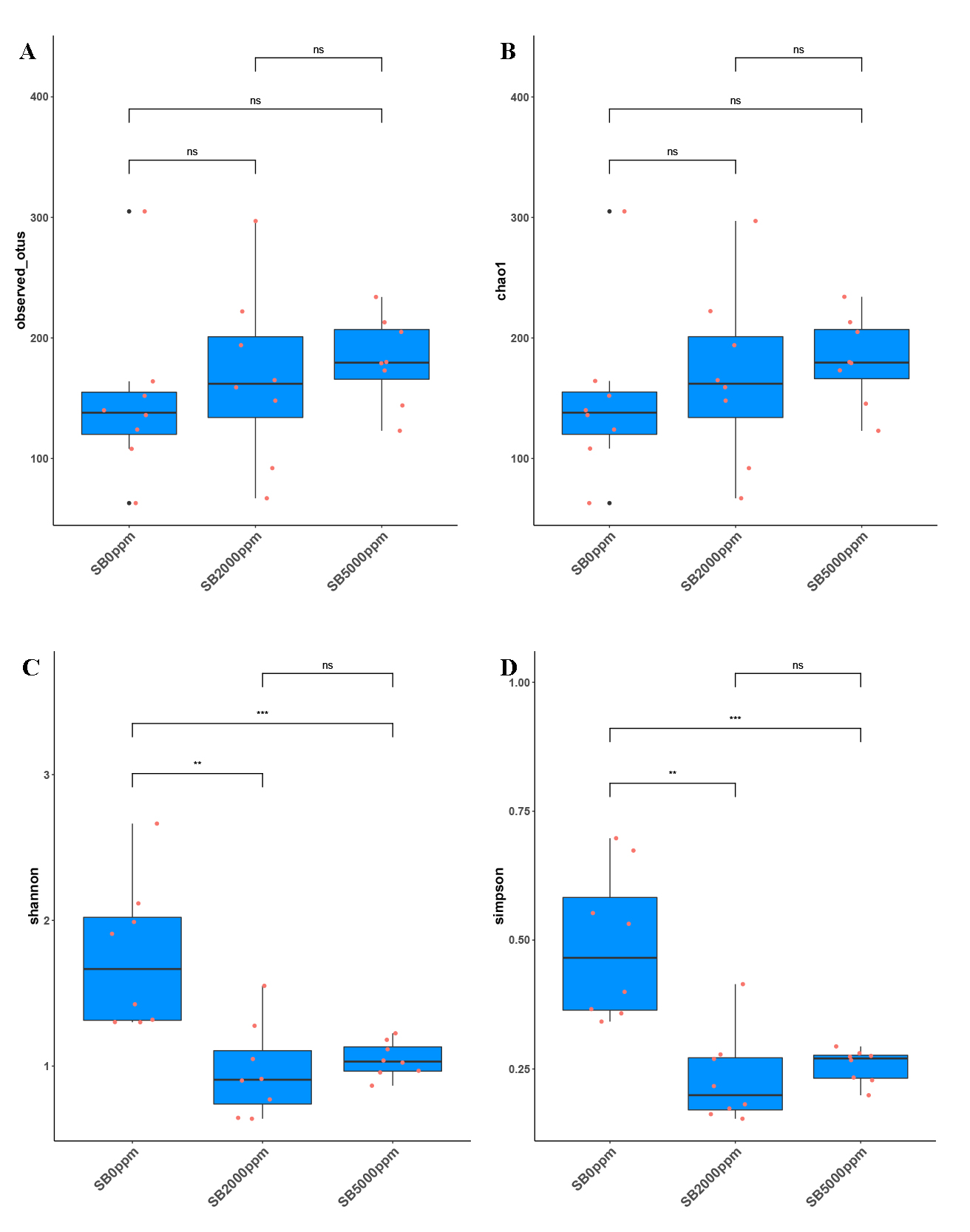

Supplement: Supplementary Figure 2 — Alpha diversity of flies’ commensal microbiota of Wolbachia-included data fed with SB at the genus level. (A) Observed OTUs. (B) Chao1. (C) Shannon. (D) Simpson. [file Image_2.JPEG]

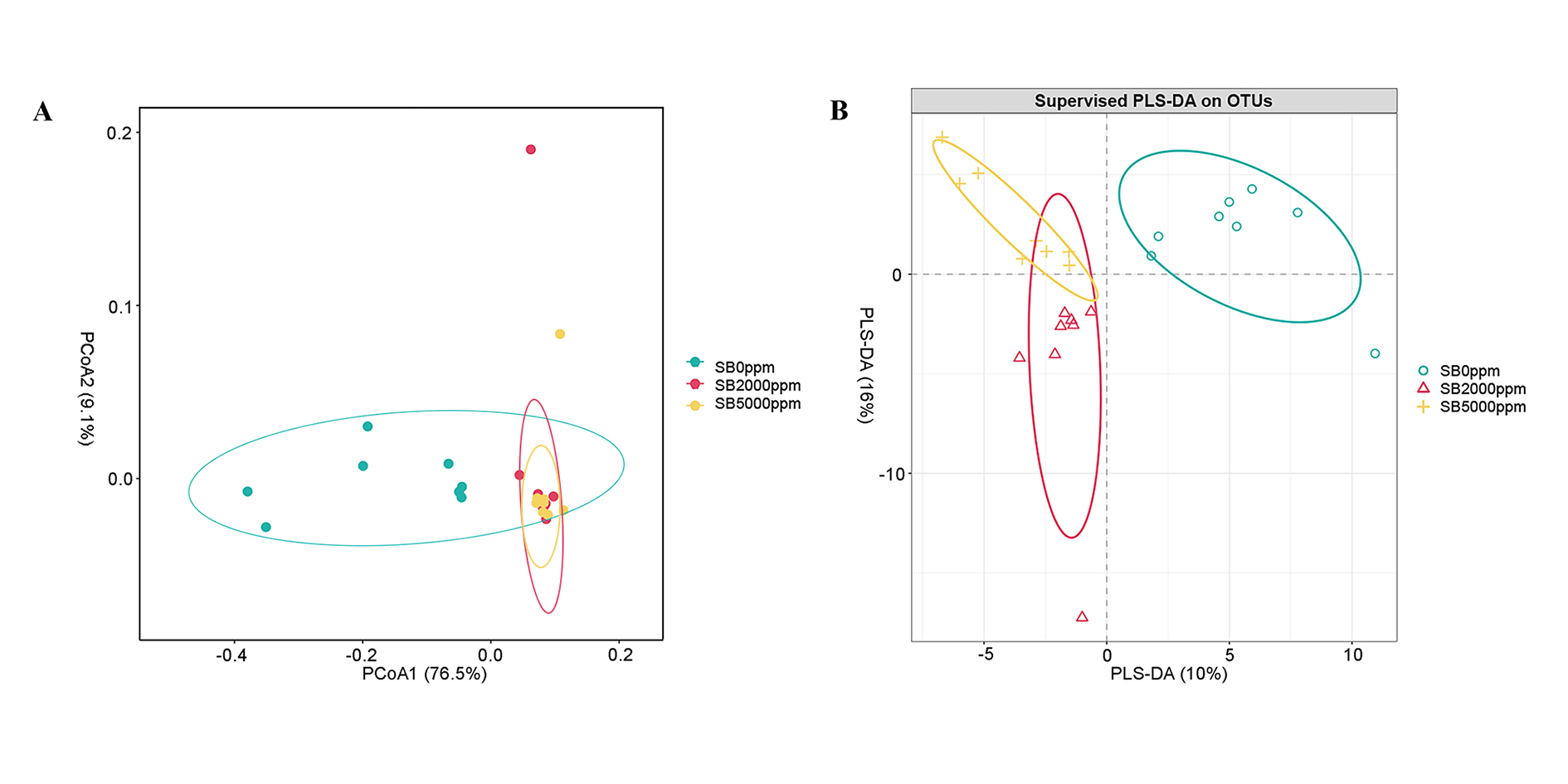

Supplement: Supplementary Figure 3 — Beta diversity of flies’ commensal microbiota of Wolbachia-included data fed with SB at the genus level. (A) Principal coordinate analysis (PCoA) score plot based on Bray-Curtis distance. (B) Partial least squares discriminant analysis (PLS-DA) plot. [file Image_3.JPEG]

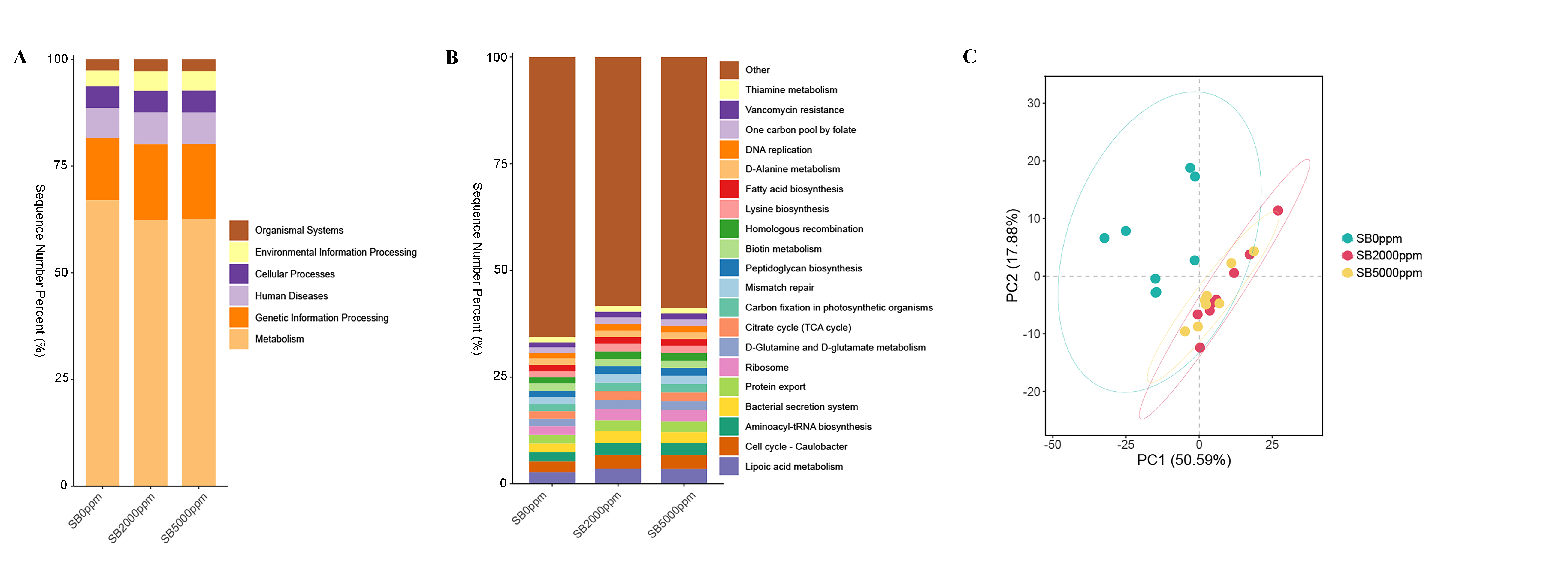

Supplement: Supplementary Figure 4 — Function prediction of commensal microbiota of Wolbachia-included data. (A) KEGG pathway at L1 level. (B) KEGG pathway at L3 level. (C) Principal component analysis (PCA) of commensal microbial function at L3 level. [file Image_4.JPEG]
